# Serum exosomal lncRNA XIST is a potential non‐invasive biomarker to diagnose recurrence of triple‐negative breast cancer

**DOI:** 10.1111/jcmm.16009

**Published:** 2021-05-05

**Authors:** Fengming Lan, Xiaodan Zhang, Huibing Li, Xiao Yue, Qinghong Sun

**Affiliations:** ^1^ Department of Radiation Oncology National Cancer Center/Cancer Hospital & Shenzhen Hospital Chinese Academy of Medical Sciences and Peking Union Medical College Shenzhen China; ^2^ Department of Radiation Oncology PLA Airforce General Hospital Beijing China; ^3^ Department of Neurosurgery Guangdong 999 Brain Hospital Guangzhou China; ^4^ Department of Neurosurgery Shenzhen University General Hospital Shenzhen China; ^5^ Shenzhen Woke Biomedical Technology Co., Ltd. Shenzhen China

**Keywords:** biomarker, lncRNA XIST, serum exosome, triple‐negative breast cancer

## Abstract

Exosomal lncRNAs secreted by cancer cells can serve as potential biomarkers in the diagnosis and prognosis of various tumours. Here, we are committed to explore the diagnostic and prognostic value of serum exosomal XIST secreted by tumour cells to predict recurrence in patients with triple‐negative breast cancer (TNBC). Significant increments in XIST and exo‐XIST from tumour tissues and blood serum were found in reoccurring TNBC patients by comparison with non‐recurrences. Levels of serum exo‐XIST were only significantly increased in TNBC recurrence and no association with other clinicopathological parameters. Additionally, serum exo‐XIST levels could be served as an assessment of change in the load of triple‐negative breast cancer. Expressions of exo‐XIST were markedly decreased after resection of the primary breast tumours and obviously elevated at the time of recurrence. Finally, an obvious association was identified between serum exo‐XIST levels and a poorer overall survival (OS) in TNBC patients. Levels of serum exo‐XIST may serve as a diagnostic and prognostic biomarker to predict the recurrent TNBC‐loading status.

## 1 INTRODUCTION

Breast cancer is confirmed as the second most frequently diagnosed cancer, however, with the leading mortality rate in females worldwide. As reported, breast cancer affects approximately 12.4% women during their whole lifetime.^1^ Breast cancer which are divided into many subtypes is a heterogeneous dependent disease, and the most common type is carcinomas. Mostly, patients with breast cancer will get more than one treatment type based on cancer type, special stage and any other situations. Triple‐negative breast cancer (TNBC), a subtype of breast cancer that is characterized by lack expression of oestrogen receptor (ER), progesterone receptor (PR) and human epidermal growth factor receptor 2 (HER2), accounting for about 15% of all breast cancers. TNBC typically presents in younger women and is clinically unique due to its aggressive and metastatic nature.^2^, ^3^ As for now, current standard therapy for TNBC includes chemotherapy, most commonly delivered before maximal surgical resection followed by systemic treatments, as there is no effect on hormonal and targeted therapy.

Despite multimodality treatment, TNBC is associated with aggressive and metastatic clinical behaviour, early recurrence and poor prognosis. About 5% of TNBC patients present with distal metastases at the time of diagnosis. This metastatic form is the most aggressive and has a poorer prognosis.^4^ The primarily metastasic organs of breast cancer are bone, lungs, lymph nodes, liver and brain.^5^ On the other hand, TNBC undergoing progression to recurring phenotype was the major cause of mortality in cancer patients. In common, the risk of recurrence remains consistent for >10 years in other breast cancer subtypes after systemic therapy. Nevertheless, patients with TNBC have high rates of recurrence in 1‐4 years, thereafter with the risk declining rapidly. Therefore, it is imperative to develop the effective treatment options for TNBC and to elucidate the molecular mechanisms of recurrence. Only a deeper identifying the potential biomarkers and novel therapeutic targets for a more precise recurrent prediction can offer hope to develop efficient therapies of TNBC.

Over the past decades, large‐scale genome and transcriptome studies obtained much more non‐coding RNAs compared with transcriptional RNA, including short non‐coding RNAs and long non‐coding RNAs (lncRNAs). LncRNAs are defined as transcripts longer than 200 nucleotides, and initially regarded as ‘junk DNA’ owing to the lack of protein‐coding function. LncRNAs constitute a heterogeneous class of RNAs, including sense, antisense, bidirectional, intronic and intergenic lncRNAs.^6^, ^7^, ^8^ However, the majority of the lncRNAs have not yet been functionally characterized to date.

## 2 MATERIALS AND METHODS

### 2.1 Patients and clinical samples

Blood serum samples were collected from 91 patients with histologically proven TNBC after operation from April 2010 to October 2013. All participants signed informed consent for biomarker identification through tissue sample collection prior to recruitment. All specimens were handled and made anonymous according to the ethical and legal standards.

All procedures performed in studies involving human participants were in accordance with the ethical standards of the institutional and/or national research committee and with the 1964 Helsinki declaration and its later amendments or comparable ethical standards.

All patients were followed up at regular intervals for up to 5 years. Twenty‐five patients incurred recurrence after long‐term following up, and the other 66 patients were divided into no recurrent group. Survival times were calculated from the diagnosis date to the decease date or the last date of follow‐up. Patients who died from other diseases unrelated to breast cancer, occurred metastasis or unexpected events were excluded from this study.

### 2.2 Isolation of exosomes from serum and conditioned media

Total exosome isolation reagent was purchased from Invitrogen to isolate exosomes according to the instructions. Briefly, serum was firstly centrifuged at 3000 g for 30 minutes, and exosome isolation reagent was added to the supernatant. After incubation, the mixture was centrifuged at 1 × 10^4^ g for 30 minutes. SDS sample buffer was last to dissolve total exosomal pellets.^9^

### 2.3 RNA isolation and Real‐Time PCR

TRIzol reagent (Invitrogen) was applied to isolate total RNA from the serum samples. The RNA was then reverse transcribed into cDNA using the SuperScript III^®^ (Invitrogen) and then amplified by RT‐qPCR based on the TaqMan method on a Bio‐Rad CFX96 Sequence Detection system (Bio‐Rad). The thermocycling condition was 95°C for 10 minutes, followed by 40 cycles of 95°C for 15 seconds and 60°C for 1 minutes. The levels of LncRNA XIST were normalized to GAPDH expression.^10^, ^11^

### 2.4 Statistical analysis

All of the statistical analyses were assessed by commercially available software SPSS version 13.0 (SPSS). The analysis of variance was performed to evaluate the statistical differences among groups. Statistical significance was determined as *P* < .05 (*) or .01 (**).

## 3 RESULTS

### 3.1 Clinical characteristics of patients

In total, 91 patients diagnosed as TNBC including 12 cases with stage group Ⅱ A, 42 cases with stage group Ⅱ B and 37 cases with stage group Ⅲ were recruited for this study from April 2010 to October 2013 according to AJCC TNM staging 7th edition.^12^ Based on long‐term following up observation, 25 patients occurred in recurrence. Therefore, all patients were divided into two groups, 66 non‐recurring TNBC and 25 non‐recurring TNBC. The median age of the patients was 53 years (range: 24‐72 years). The median Karnofsky Performance Status (KPS) score was 90 (range: 70‐100). None of the patients received chemotherapy or radiotherapy prior to surgery. All patients received radio‐chemotherapy after surgery. The median follow‐up time was 39 months (range: 11‐62 months). Overall, 29.7% of participants were died due to the local recurrence of TNBC confirmed by pathologically. At the time of the last follow‐up, 27 patients were deceased, including 2 patients from the stage IIA group, 8 patients from the stage IIB group and 17 patients from the stage Ⅲ group. The duration from tumour recurrence to the death date was not very long for TNBC patients (median 9 months; 0‐26). The 1‐year, 2‐year and 5‐year overall survival (OS) rates were 100%, 86.3% and 70.3%, respectively.

### 3.2 Overexpression of XIST in human tumour tissues and serum exosome in recurrent TNBC patients

**FIGURE 1:**
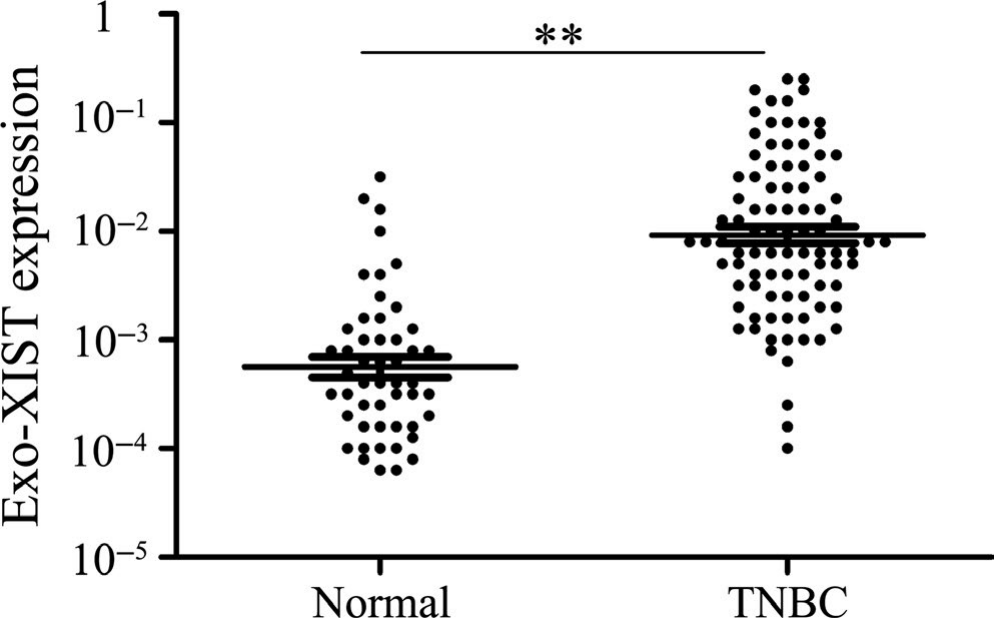
Serum exosomal XIST expression in patients with TNBC and healthy controls analysed by qRT‐PCR. **P < .01

**FIGURE 2:**
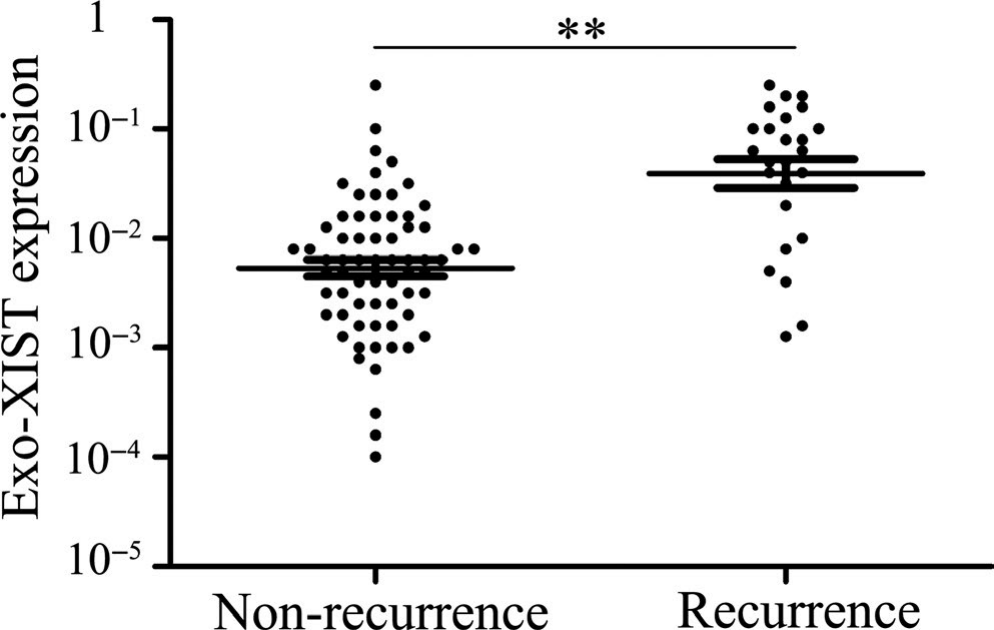
Serum exosomal XIST expression in recurrence and non‐recurrence TNBC patients

Initially, qPCR was used to examine the expression of lncRNA XIST in serum. Levels of serum exo‐XIST were analysed to compare the difference between TNBC patients (n = 91) and healthy controls (n = 50) by qPCR. There is an obviously significant difference in the expression of serum exo‐XIST between TNBC patients and health control after normalization (Figure 1). Importantly, expressions of exosomal XIST were extracted from serum before the resection of tumours in breast cancer patients and analysed to compare the relapses or not by using scatter plots. As the results in tissues' expression, there exists a significant difference in exo‐XIST expression between recurrent and non‐recurrent TNBC patients, and XIST exosomes were significantly higher in the recrudescent TNBC patients (*P* < .01, Figure 2).

### 3.3 Exo‐XIST expression levels are associated with clinicopathological features

Next, we explored supposed associations between levels of serum exo‐XIST expression and various clinicopathological features. To this end, median expression level was used as a cut‐off to the divide 91 TNBC patients into low and high exo‐XIST expression groups. The percentages of patients with stage Ⅱ A, stage Ⅱ B and stage Ⅲ were 46.7%, 53.1% and 56.0% in the high exo‐XIST group, respectively. There was no statistical relationship between expression of serum exo‐XIST and different stages. There were obviously significant correlations between high serum XIST expression levels and recurrent TNBC (Table 1). No association was observed between the serum exo‐XIST levels and any of the other clinicopathological parameters tested, including KPS, gender and age at diagnosis (*P* > .05, Table 1).

**TABLE 1:** Correlation between clinical characteristics and exosomal XIST level

Parameter	Patients (n)	Exosomal XIST	
		Mean expression	*P* value
Status			
Recurrence	25	0.0732	<.01
Non‐recurrence	66	0.0075	
Age			
≤53	40	0.0469	.579
>53	51	0.0345	
Stage			
II	54	0.0547	.621
III	37	0.0321	
KPS			
≤90	27	0.0405	.532
>90	64	0.0431	

### 3.4 Serum levels of exo‐XIST reflect tumour burden in matched TNBC samples

**FIGURE 3:**
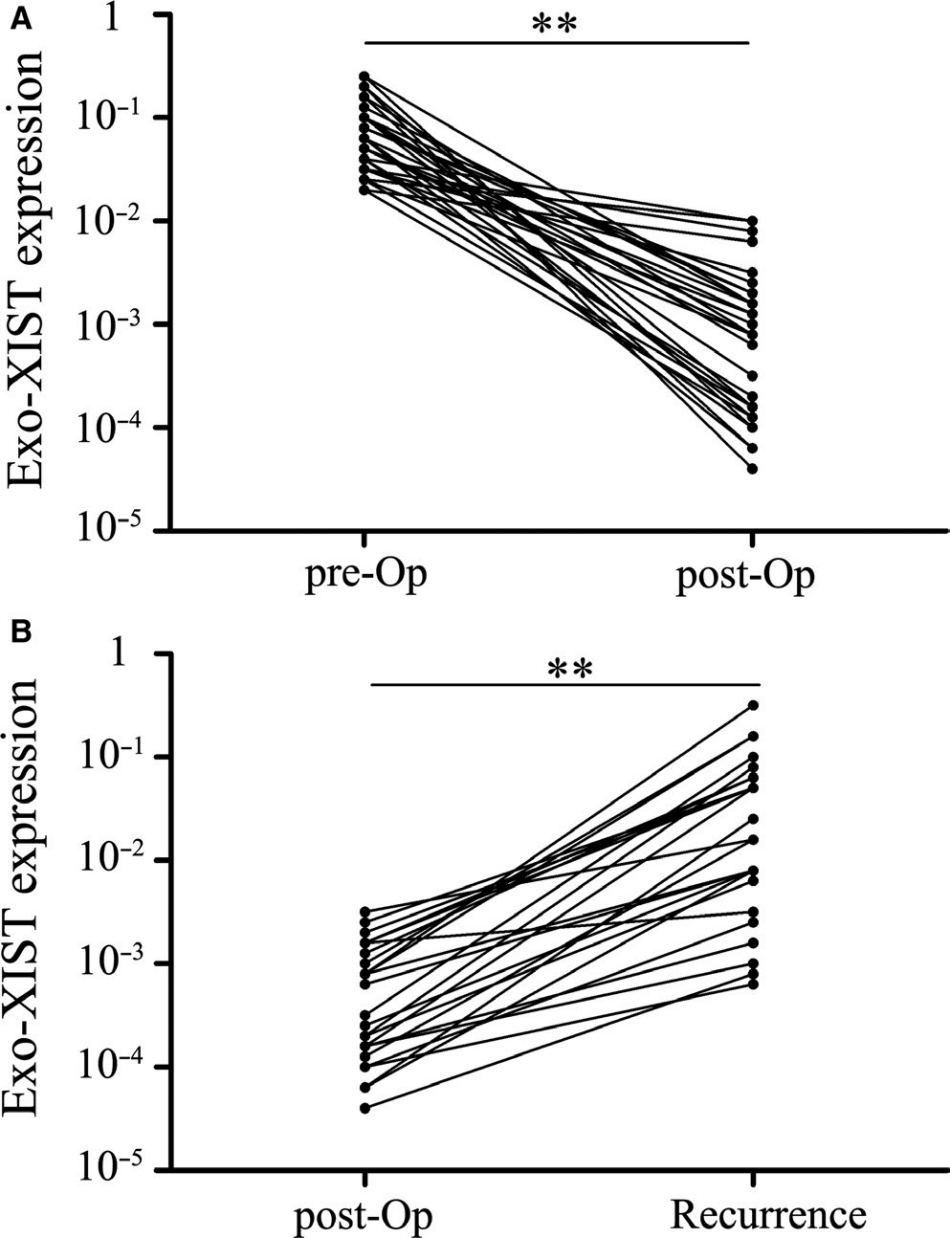
Serum exosomal XIST levels reflect tumour dynamics. A, Serum exosomal XIST expression levels before and after surgery. The XIST levels are significantly lower in the post‐operative samples than in the pre‐operative samples (**P < .01). B, Comparison of serum exosomal XIST levels in samples obtained post‐operatively and after recurrence. A significant increase in serum exosomal XIST levels is found at recurrence. **P < .01

Firstly, the serum exo‐XIST was extracted from 30 TNBC patients in pre‐ and post‐operation and examined to identify the changes or not. Interestingly, it has been observed that serum exo‐XIST levels markedly decreased after resection of the primary breast tumours (Figure 3A). Additionally, 25 TNBC recurrent patients included to compare the expressions of serum exo‐XIST after complete surgery and the recurrence of breast tumour. The exo‐XIST levels were still detectable at relatively low levels after surgery, but significantly increased at the time of recurrence (Figure 3B). Taken together, serum exo‐XIST which could reflect tumour burden during treatment may be specifically secreted and released by TNBC cells.

### 3.5 Serum exo‐XIST expression as a potential diagnostic biomarker for TNBC patients

**FIGURE 4:**
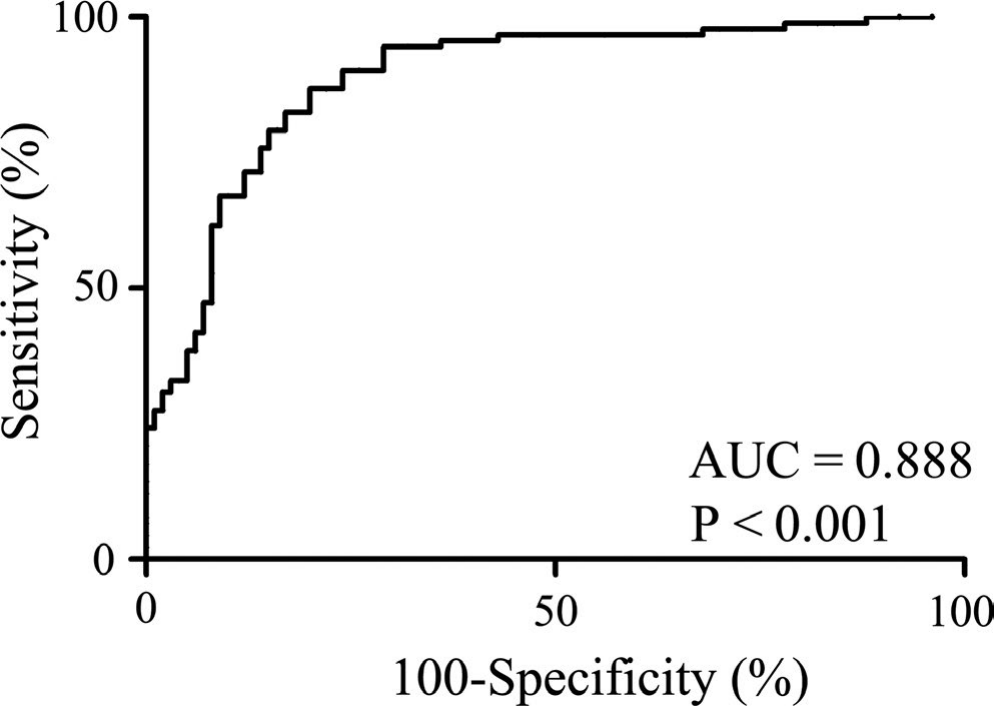
ROC curve analysis based on serum exosomal XIST levels for distinguishing TNBC patients from normal controls

To evaluate the diagnostic value of serum exo‐XIST expression for TNBC patients, ROC curve analysis and the corresponding AUC values were applied to evaluate the diagnostic potential and discriminatory accuracy of serum exo‐XIST. ROC analyses revealed that serum exo‐XIST levels were robust in discriminating patients with TNBC from healthy control, with an AUC value of 0.888 (95% CI: 0.814‐0.935), whereas the sensitivity, specificity, and positive and negative predictive values to identify patients with TNBC were 84.2%, 92.3%, 93.8% and 71.2%, respectively (Figure 4).

### 3.6 Association between serum exo‐XIST expression and prognosis of TNBC patients

**FIGURE 5:**
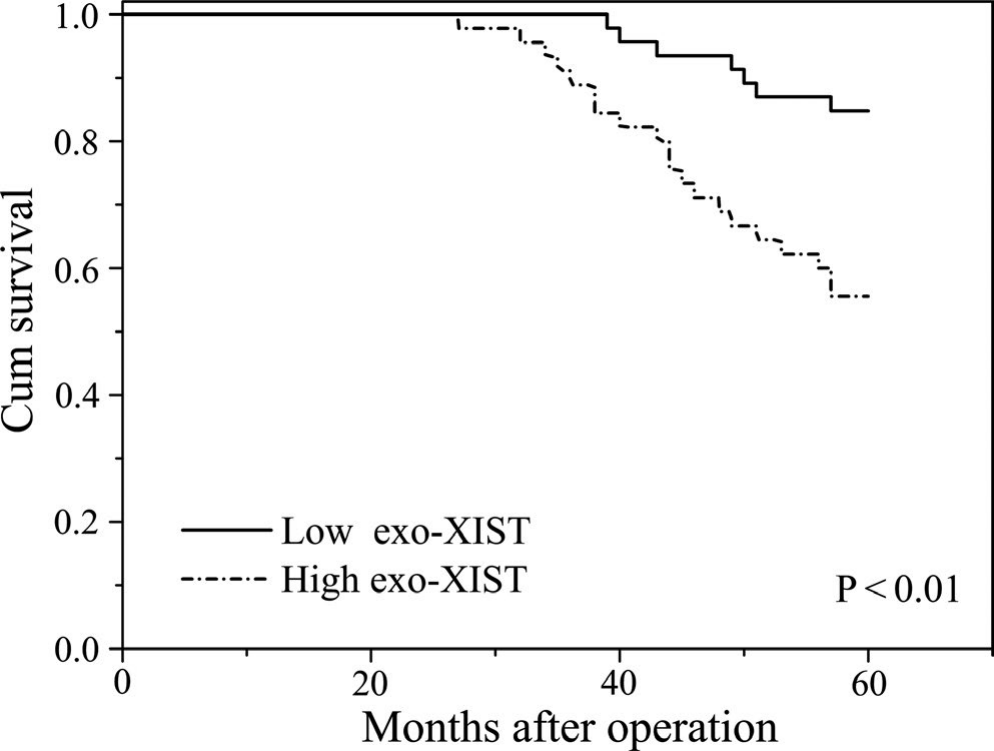
Kaplan‐Meier survival curves for patients with TNBC and high or low serum exosomal XIST expression levels

In this cohort, subsequent Kaplan‐Meier analysis was used to demonstrate the prognostic function of exo‐XIST in TNBC patients. As revealed in Figure 5, patients with a low serum exo‐XIST expression have a relatively higher OS rates than that of patients with an increasing level of exo‐XIST. In order to additionally assess the prognostic value of serum exo‐XIST levels for the overall survival, Cox regression analyses were carried out to assess associations between prognostic factors and serum exo‐XIST expressions. By doing so, a significant association was identified between serum exo‐XIST levels and OS in TNBC patients (*P* < .01, hazard ratio [HR] 3.54, 95% confidence interval [CI] (1.55‐5.45)). Collectively, we certified that the levels of serum exo**‐**XIST could function as an excellent factor to indicate the recurrence TNBC.

## 4 DISCUSSION

Recently, lncRNAs have proven to be a new patient of innovation and research focus by comparison with mRNAs and protein molecules.^13^ The aberrantly expressed lncRNAs have been found to be implicated in tumorigenesis and development of various cancers.^14^ Increasing evidences revealed that lncRNAs were contained in a series of gene expression and then participated in biological processes at cell cycle, proliferation, apoptosis, invasion and migration. As reported, lncRNAs included a mass of molecular bioinformatics as they involved in the largest portion in post‐transcription, transcription and epigenetic regulation.^15^ Nowadays, lncRNAs are emerging as crucial biomarker in the diagnosis and prognosis of various cancers. ABHD11‐AS1 was abnormally up‐regulated in pancreatic cancer and played an important role in predicting prognosis.^16^ LncRNA associated with TF efficacy can serve as the prognostic and therapeutic biomarkers in ovarian cancer.^17^ LncRNA‐LINP1 is a novel non‐invasive factor to predict the prognosis of breast cancer.^18^ FER1L4 is an independent indicator of poor prognosis in endometrial carcinoma.^19^

Exosomes are nanovesicles, length in 30‐140 nm, carrying genetic factors such as mRNAs, proteins, lncRNAs and miRNAs. More and more researches displayed that lncRNAs which are packaged by exosome secreted from various cell types including cancer cells. LncRNAs contained in exosomes are obviously stable and recognized as promising biomarkers in diagnosis and prognosis of cancers. Microarray analysis and qRT‐PCR identified six increased exo‐lncRNAs from plasma of colorectal cancer and proved to be potential biomarkers in early diagnosis.^20^ Serum exosomal H19 is identified as a potentially diagnostic and prognostic biomarker in bladder cancer.^21^ LncRNAs extracted from urinary exosomes are functioned as potentially diagnostic indicators in bladder cancer.^22^ Serum exo‐lncRNAs can diagnose and predict recurrence in bladder cancer.^23^ Circulating exo‐lncRNAs from serum appear to be promising biomarkers to suggest the prognosis of human hepatocellular carcinoma.^24^

In this study, we are aimed to explore whether exo‐lncRNA XIST is a potentially non‐invasive biomarker to predict the recurrence and prognosis of TNBC patients. Firstly, Significant increases in XIST and exo‐XIST from tumour tissues and serum were found in reoccurring TNBC patients by comparison with non‐recurrences. Levels of serum exo‐XIST only significantly increased in TNBC recurrence and no association with other clinicopathological parameters. Additionally, serum exo‐XIST levels could be served as an assessment of change in breast cancer load. Expressions of exo‐XIST were markedly decreased after resection of the primary breast tumours, and obviously increased at the time of recurrence. Finally, a significant association was identified between serum exo‐XIST levels and a poorer overall survival (OS) in TNBC patients.

In summary, serum exosomal XIST is abnormally elevated in recurrent TNBC patients and can function as a novel non‐invasive biomarker to predict the progress of TNBC patients. Certainly, the molecular mechanisms of underlying remain largely unknown and further supporting evidences is in need from larger independent studies before applying to clinical practice.

## CONFLICT OF INTEREST

The authors declare that they have no conflict of interest.

## AUTHOR CONTRIBUTION

**FengMing Lan:** Data curation (equal); Resources (equal); Writing‐original draft (equal). **Xiaodan Zhang:** Formal analysis (equal); Validation (equal). **Huibing Li:** Formal analysis (equal); Validation (equal). **Xiao Yue:** Conceptualization (equal); Resources (equal); Writing‐original draft (equal). **Qinghong Sun:** Conceptualization (equal); Software (equal); Writing‐review & editing (equal).
